# A Young Boy with Neck Pain

**DOI:** 10.5811/cpcem.2020.12.50242

**Published:** 2021-03-24

**Authors:** Hirofumi Ohno, Shinsuke Takeda, So Mitsuya, Hisatake Yoshihara, Ken-ichi Yamauchi

**Affiliations:** * Toyohashi Municipal Hospital, Department of Orthopedic Surgery, Toyohashi, Japan; † Toyohashi Municipal Hospital, Department of Trauma and Microsurgery, Toyohashi, Japan; ‡ Toyohashi Municipal Hospital, Department of Spine Surgery, Toyohashi, Japan

**Keywords:** Pediatric idiopathic intervertebral disk calcification, neck pain, PIIVDC calcification

## Abstract

**Case Presentation:**

A five-year-old boy presented to our emergency department with severe posterior neck pain that was exacerbated upon neck movement. Cervical spine radiography revealed calcification in the cervical intervertebral disk 3–4.

**Discussion:**

Pediatric idiopathic intervertebral disk calcification is a benign, rare condition that might be complicated by associated severe neurological symptoms. In this case, the symptoms gradually subsided with conservative management alone.

## CASE PRESENTATION

A five-year-old male with a history of asthma presented with complaints of neck pain that had persisted for one month. The pain exacerbated on bowing or exercising. Physical examination revealed that pain was elicited on performing neck movements, especially flexion-extension; neurological symptoms were not noted. Cervical spine radiograph was obtained (Image).

## DISCUSSION

Pediatric idiopathic intervertebral disk calcification (PIIVDC) is a rare cause of neck pain in children, especially boys, 5–12 years of age, and is attributed to calcification of the intervertebral disks.^1^,^2^ Since its first description in 1924, more than 300 cases of PIIVDC have been reported, but it is not well known to emergency physicians. PIIVDC can affect any spinal cord region, although it mostly affects the cervical spine, especially the cervical intervertebral disk 3–4 or 6–7.^1^ The etiology of PIIVDC remains unclear. Trauma, infection, nutritional supply, metabolic disorders, and hereditary deficit may be related to PIIVDC.^2^ The most common clinical symptoms of PIIVDC are neck pain and stiffness followed by muscle spasm, low-grade fever, radiating pain, and torticollis due to local inflammation of the nucleus pulposus.^1^,^2^

Neurological complications develop when the calcification herniates and compresses the nerve root or spinal cord.^1^ Conservative management, including administration of analgesics, nonsteroidal anti-inflammatory medication, muscle relaxants, use of cervical soft collars, and rest, is preferred in most cases because the symptoms are generally self-limiting and resolve within a few months. The calcified lesions may remain asymptomatic for some time.^1^,^2^ If neurological impairment progresses, surgical treatment, such as laminectomy or discectomy and fusion, should be considered.^3^ In our case, the symptoms gradually improved with conservative treatment. Physicians involved in emergency care should consider PIIVDC during the differential diagnosis of children with neck pain.

**CPC-EM Capsule**

What do we already know about this clinical entity?

*Pediatric idiopathic intervertebral disk calcification (PIIVDC) is a benign, rare condition with unclear etiology.*

What is the major impact of the image(s)?

*PIIVDC is easily diagnosed with a plain radiograph. We should consider cervical spine radiograph as the initial test for children with neck pain.*

How might this improve emergency medicine practice?

*Emergency physicians should consider PIIVDC in their differential diagnosis of children with neck pain.*

## Figures and Tables

**Image:**
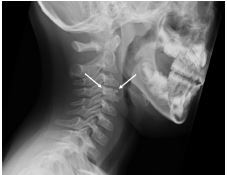
Cervical spine lateral radiograph showing the calcification in the cervical intervertebral disk 3–4 (arrow). This radiological abnormality is not clearly visible on an anterior-posterior view.
